# The effect of synbiotic preparations on the intestinal microbiota and her metabolism in broiler chickens

**DOI:** 10.1038/s41598-020-61256-z

**Published:** 2020-03-09

**Authors:** Katarzyna Śliżewska, Paulina Markowiak-Kopeć, Artur Żbikowski, Piotr Szeleszczuk

**Affiliations:** 1 Institute of Fermentation Technology and Microbiology, Faculty of Biotechnology and Food Sciences, Lodz University of Technology, Wólczańska 171/173, 90-924 Lodz, Poland; 2 Department of Pathology and Veterinary Diagnostics, Faculty of Veterinary Medicine, Warsaw University of Life Sciences – SGGW, Nowoursynowska 159c St., 02-776 Warsaw, Poland

**Keywords:** Animal biotechnology, Applied microbiology

## Abstract

The aim of the research was to determine the effect of newly elaborated synbiotic preparations on the count of dominant intestinal microorganisms, on the profile of fatty acids (short chain – SCFA and branched chain – BCFA), the lactic acid produced and the performance of chickens. The studies determined the composition of the dominant intestinal microbiota with use of the culture method. The fatty acid profile was also determined using the high-performance liquid chromatography method (HPLC). Moreover, the performance of chickens was determined such as the daily cumulative mortality rate, the feed conversion ratio (FCR) and the European Production Efficiency Factor (EPEF). It was found that synbiotics had a beneficial effect on parameters of the performance of chickens, and also resulted in increase in the count of beneficial bacteria and to the restriction in growth of potential pathogens in the gastrointestinal tract. Synbiotics caused an increase in the concentration of lactic acid and SCFA and a decrease in the concentration of BCFA in the broiler’s excreta. These results showed a beneficial effect of the tested synbiotics on the intestinal microbiota, their metabolism and the performance of broiler chickens. The elaborated synbiotics can be successfully used as feed additives for broiler chickens.

## Introduction

The balance among the gastrointestinal microbiota is an important factor affecting digestion, absorption of nutrients and animal health. The gut is also a major site of potential exposure to environmental pathogens^1^. Moreover, a well-functioning and healthy gut is the cornerstone of the optimum performances of the birds^2^. The development of the intestinal microbiota starts at hatching, when chickens get bacteria, inter alia, from the surface of the eggshell or directly from the mother^3,4^. The composition of the gut microbiota has been found to be affected by multiple factors such as diet, age, genotype, sex, hatching condition, litter, and feed management^5,6^. The composition of the intestinal microbiota is readily changeable, which favours the development of gut microorganisms-targeted therapies such as antibiotics, prebiotics, probiotics and synbiotics^7^. Long-term use of antibiotics has led to the development of drug-resistant microorganisms, posing a threat to consumers’ and animals’ health and also exerting a negative effect on the environment^8^. As a result, the use of antibiotic-based growth stimulators was banned in the European Union on 1 January 2006^9^. Prebiotics, probiotics, and synbiotics are alternative feed additives for the banned antibiotic-based stimulators^10^.

According to the definition formulated in 2002 by WHO (World Health Organization) and FAO (Food and Agriculture Organization of the United Nations), probiotics are ‘live microorganisms which, when administered in sufficient amounts, confer a health benefit on the host’^11^. This definition was maintained in 2013 by the International Scientific Association for Probiotics and Prebiotics (ISAPP) and is still presently used^12^. In 2007, WHO/FAO experts defined prebiotics as ‘a nonviable food component that confers a health benefit on the host associated with modulation of the microbiota’^13^. Prebiotics may be used independently or as an additional support for to probiotic microorganisms. However, various prebiotics stimulate the growth of different native intestinal bacteria^14^. Formulas containing both synergistically acting prebiotics and probiotics are already used in nutrition; they are called ‘synbiotics’.

The quantitative and qualitative composition of microbiota in various sections of the gastrointestinal tract may change under the influence the zoohygenic conditions of the environment and particularly the composition of the feed administered. In addition, changes can also be seen in the profile of metabolites produced by intestinal microorganisms, e.g. fatty acids or lactic acid.

In recent years, the beneficial effects of synbiotics on the health of broiler chickens have been repeatedly confirmed, e.g. in preventing the negative effects of heat shock, particularly when exposed to hot climates. Studies conducted on broiler chickens exposed to cyclic heat stress confirm the positive effect of the synbiotic (PoultryStar meUS) on the changes in the intestinal histomorphology and expressions of heat shock protein70 (HSP70) in tested animals^15^. In other study carried out on chickens given a synbiotic (0.8% of the prebiotic RFO (extracted from lupine seeds) and 1% LAVIPAN) together with feed, improvement of the intestinal morphometric parameters of broiler chickens was confirmed^16^. The health effect of synbiotics is probably associated with the individual combination of a probiotic and prebiotic^17^. Significant health benefits and in terms of chicken performance have been found in studies based on the use of probiotic (Protexin), prebiotic (Immunoval) and synbiotic (Biomin IMBO) compared to antibiotic (Flovomycin) and control treatment. The best results were obtained with the synbiotic but specific benefits of the prebiotic in reducing blood cholesterol and increasing lactic acid producing bacteria were observed^18^. In addition, recent report indicate that early *in ovo* treatment of chicken embryos with synbiotics and probiotics may temporarily modulate the production/maturation of leukocytes and their reactivity^19^. These findings confirm the beneficial effects of the bioactive substances tested on the innate immune system of chickens.

Due to the high effectiveness of synbiotics in animal nutrition and the demand for such preparations, prototypes of three new synbiotic preparations for monogastric animals were developed. Lactic bacteria of *Lactobacillus* and yeast of *Saccharomyces cerevisiae* are QPS (Qualified Presumption of Safety) microorganisms and have high efficiency in feeding monogastric animals^10^. So, in the composition of the newly elaborated synbiotic preparations, *Lactobacillus* spp. and *Saccharomyces cerevisiae* strains and inulin (as prebiotic) were used. These strains were isolated from various sources (*Lb. paracasei* ŁOCK 1091 - caecal content of sow; *Lb. pentosus* ŁOCK 1094 - broiler chicken dung; *Lb. plantarum* ŁOCK 0860 - plant silage; *Lb. reuteri* ŁOCK 1092 - piglet caecal content; *Lb. rhamnosus* ŁOCK 1087 – turkey dung and *S. cerevisiae* ŁOCK 0119 – distillers’ yeast, grain). Strains were studied within the PBS3/A8/32/2015 project and have been deposited in the Lodz Collection of Pure Cultures (ŁOCK 105) of the Institute of Fermentation Technology and Microbiology, Lodz University of Technology (Poland) and also in the Polish Collection of Microorganisms (PCM) of the Institute of Immunology and Experimental Therapy, Polish Academy of Sciences (Poland). The strains from the elaborated synbiotic preparations possess full probiotic documentation described in patent applications and the patent description^20–25^. Research results on antagonism of pathogens, adherence to Caco-2, inhibition of adherence of pathogens to Caco-2, antibiotic resistance, resistance to bile salts and low pH, auto- and coaggregation and hydrophobicity are being published (authors: Śliżewska K. and Chlebicz A.). Moreover, additional information about beneficial activities of probiotic strains contained in the synbiotics’ composition is available in publications about research *in vitro* and *in vivo* (Table 1)^26–28^. The comparison of the effect of the newly elaborated preparations with commercial preparations is very important. So, probiotic preparations contained *B. licheniformis* DSM 5749 and *B. subtilis* DSM 5750 (BioPlus YC; Biochem) and *Enterococcus faecium* NCIMB 10415 (SF68) (Cylactin; DSM) were also used in the research. The effectiveness of *B. licheniformis* DSM 5749 and *B. subtilis* DSM 5750 was confirmed *in vitro*^29^ and in studies on chickens^30^, sows^31^, and lambs^32^, while a strain of *Enterococcus faecium* NCIMB 10415 (SF68) was the subject of research *in vitro*^24^ and research in piglets, sows^33–35^, chickens^36^.

**Table 1:** Information about probiotic strains from the elaborated synbiotic preparations and commercial probiotics.

Microorganism	Collection number	Origin	Beneficial activities of strain (reported *in vitro*)
*Lb. paracasei*	ŁOCK 1091	Caecal content of sow	∙ antagonism of pathogens, adherence to Caco-2, inhibition of adherence of pathogens to Caco-2, antibiotic resistance, resistance to bile salts and low pH, auto- and coaggregation and hydrophobicity^14–19^;
*Lb. pentosus*	ŁOCK 1094	Broiler chicken dung	
*Lb. plantarum*	ŁOCK 0860	Plant silage	
*Lb. reuteri*	ŁOCK 1092	Piglet caecal content	
*Lb. rhamnosus*	ŁOCK 1087	Turkey dung	
*S. cerevisiae*	ŁOCK 0119	Distillers’ yeast, grain	
			∙ ability to detoxify of aflatoxin B1, deoxynivalenol, fumonisins, T-2 toxin and zearalenone^20^;
			∙ ability to detoxify ochratoxin A by reducing its concentration and genotoxicity^21^.
*B. licheniformis*	DSM 5749	Soil	∙ safety and efficacy of *B. licheniformis* DSM 5749 and *B. subtilis* DSM 5750 has been confirmed in studies *in vitro*^23^.
*B. subtilis*	DSM 5750	Soybean fermentation	
*E. faecium*	NCIMB 10415 (SF68)	Faeces of a healthy breast-fed new-born baby (Asplund, 1991)	∙ safety and efficacy of *E. faecium* NCIMB 10415 (SF68) has been confirmed in studies *in vitro*^24^.

The aim of the research was to determine the effect of the newly elaborated synbiotic preparations, used as an addition to feed, on the count of dominant intestinal microbiota in chickens, on the profile of fatty acids (SCFA and BCFA), lactic acid produced and the performance of animals. The use of probiotic microorganisms isolated from the digestive system of monogastric animals, comparison of the elaborated preparations with commercially available formulas as well as the use of a representative large number of animals and a wide range of analysis are aspects confirming the novelty of the research compare to other similar studies.

## Materials and Methods

### Probiotic and synbiotic formulas

Synbiotic preparations (A, B or C) were elaborated in the Institute of Fermentation Technology and Microbiology of the Lodz University of Technology (Poland). Each synbiotic preparation comprised 2 × 10^9^ CFU g^−1^ LAB of *Lactobacillus* spp., 2 × 10^7^ CFU g^−1^ of *Saccharomyces cerevisiae* yeast and 2% inulin (prebiotic)^28^. Commercial probiotic preparations contained 1.6 × 10^9^ CFU g^−1^ *Bacillus* spp. such as *B. licheniformis* DSM 5749 and *B. subtilis* DSM 5750 (BioPlus YC; Biochem), and 1.0 × 10^10^ CFU g^−1^ *Enterococcus faecium* NCIMB 10415 (SF68) (Cylactin; DSM) respectively (Table 2).

**Table 2:** Strains applied in the tested synbiotic preparations and commercial probiotic preparations.

Type of preparation	Name of preparation	Probiotic microorganisms	Beneficial activities of preparation (reported *in vivo*)
Synbiotics	A	*Lb. plantarum* ŁOCK 0860 *Lb. reuteri* ŁOCK 1092 *Lb. pentosus* ŁOCK 1094 *S. cerevisiae* ŁOCK 0119	∙ Beneficial effect of the synbiotics on the gastrointestinal tract of animals. Synbiotics composed of four and five probiotic strains decreased FW genotoxicity of chicks, after exposure to OTA, to the level seen in the control group and were more effective than synbiotics composed of three probiotic strains^21^.
	B	*Lb. plantarum* ŁOCK 0860 *Lb. reuteri* ŁOCK 1092 *Lb. pentosus* ŁOCK 1094 *Lb. rhamnosus* ŁOCK 1087 *S. cerevisiae* ŁOCK 0119	
	C	*Lb. plantarum* ŁOCK 0860 *Lb. reuteri* ŁOCK 1092 *Lb. pentosus* ŁOCK 1094 *Lb. rhamnosus* ŁOCK 1087 *Lb. paracasei* ŁOCK 1091 *S. cerevisiae* ŁOCK 0119	
			∙ Synbiotics met the basic requirements for this type of formula regarding the safety of use and had a positive effect on the health of chickens^22^.
Probiotics	BioPlus YC	*B. licheniformis* DSM 5749 *B. subtilis* DSM 5750	∙ Safety and efficacy of *B. licheniformis* DSM 5749 and *B. subtilis* DSM 5750 has been confirmed in many studies *in vivo*^25–27^.
	Cylactin	*E. faecium* NCIMB 10415 (SF68)	∙ Safety and efficacy of *E. faecium* NCIMB 10415 (SF68) has been confirmed in many studies *in vivo*^24,28–31^.

### Animal treatment

The research was conducted on 504 chickens (ROSS 308 breed). Chickens were housed in standard environmental conditions in separate cages (84 birds per cage) in one room of an animal experimental laboratory^27^. For the examination period, from day 1 to 42 of life, in each group, birds were administered synbiotic preparation A, B or C in a dose of 0.5 g kg^−1^ of feed and commercial probiotic preparation BioPlus YC or Cylactin in a dose of 0.4 g kg^−1^ or 0.035 g kg^−1^ of feed *ad libitum* respectively. The negative control was a group of birds to which feed was administrated *ad libitum* without additives. Probiotic microorganisms used in synbiotic preparations were tested under procedures recommended by FAO/WHO and EFSA. All complete dietetic compounds used in feed for chickens (starter, grower, finisher) were coccidiostats-free (EKOPLON, Poland)^28^. Detailed parameters of the applied feed are presented in Table S1.

**Figure 1:**
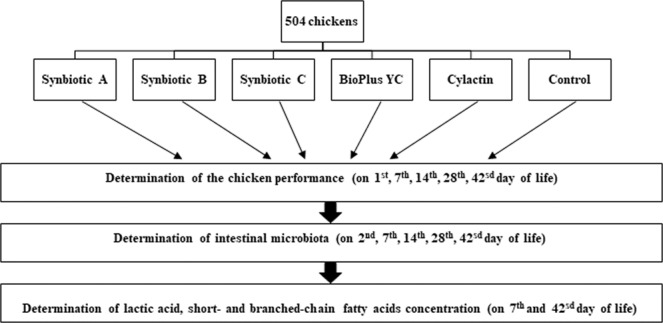
An outline of the conducted research.

Birds were grown in the Department of Avian Diseases at Department of Veterinary Pathology and Diagnostics at the Faculty of Veterinary Medicine in Warsaw University of Life Sciences (SGGW, Poland). Experiments were conducted after obtaining the approval of III Local Ethical Commission for animal testing in SGGW according to resolution No. 3/2015 from 22 January 2015^28^. All experiments were performed in accordance with the appropriate guidelines and regulations. An outline of the conducted research is presented in Fig. 1.

### Determination of the chicken performance

The chickens’ rearing time was 42 days. Chickens were observed daily in order to detect potential undesirable effects or deaths of birds. The body weight of animals in individual study groups was determined on the first and the 7^th^, 14^th^, 28^th^ and 42^sd^ day of life. Final production parameters such as daily cumulative mortality rate (%), feed conversion ratio (FCR), and European Production Efficiency Factor (EPEF) were determined according to the formulas^27^:

$${\rm{daily}}\,{\rm{cumulative}}\,{\rm{mortality}}\,{\rm{rate}}\,( \% )=\frac{{\rm{the}}\,{\rm{number}}\,{\rm{of}}\,{\rm{death}}\,{\rm{chickens}}\,({\rm{pc}}.\,)\times 100 \% }{{\rm{the}}\,{\rm{number}}\,{\rm{of}}\,{\rm{chickens}}\,{\rm{in}}\,{\rm{research}}\,({\rm{pc}}.\,)}$$

$${\rm{FCR}}=\frac{{\rm{the}}\,{\rm{feed}}\,{\rm{consumption}}\,({\rm{kg}})}{{\rm{the}}\,{\rm{body}}\,{\rm{weight}}\,{\rm{gain}}\,({\rm{kg}})}$$

$${\rm{EPEF}}=\frac{{\rm{the}}\,{\rm{liveability}}\,( \% )\times {\rm{the}}\,{\rm{body}}\,{\rm{weight}}\,({\rm{kg}})\times 100}{{\rm{the}}\,{\rm{age}}\,({\rm{days}})\times {\rm{the}}\,{\rm{feed}}\,{\rm{conversion}}\,{\rm{ratio}}\,({\rm{kg}})}$$

### Determination of intestinal microbiota

The composition of microbiota in intestinal content (the jejunum as part of the small intestine and the caecum) and in the excreta was determined on the 2^nd^, 7^th^, 14^th^, 28^th^, and 42^nd^ day of rearing in seven randomly selected chickens in each experimental group. The count of analyzed microorganisms was determined using the culture method in accordance with the PN-ISO standards in triplicate, using selective microbial media^28^. The total anaerobic bacterial count (PCA, Merck), *Enterobacteriaceae* family bacteria count (VRBD, Merck), *Escherichia coli* count (TBX, Merck) and the count of bacteria belonging to the genes *Lactobacillus* (MRS, Merck), *Bifidobacterium* (RCA), *Clostridium* (TSC with D-cycloserine, Merck), *Enterococcus* (BAA, Merck), *Bacteroides* (VL, Merck) were determined. Considering the presence of yeast in the composition of synbiotic preparations, the yeast count was also determined on SDA (Merck). Plates were incubated in conditions appropriate for a given group of microorganisms: unlimited oxygen at 37 °C for 48 hours (*Lactobacillus*, *Enterococcus*, *Enterobacteriaceae*), 44 °C for 48 hours (*Escherichia coli*), 30 °C for five days (total yeast count), and in limited oxygen at 37 °C for 48 hours (total anaerobic count, *Bifidobacterium*, *Bacteroides* and *Clostridium*)^28^.

### Determination of lactic acid and fatty acid concentrations

The level of lactic acid, short- (acetic, propionic, butyric, valeric, formic) and branched-chain fatty acids (isobutyric, isovaleric) on the 7^th^ and 42^nd^ day of rearing in the chicken excreta was determined. In the research, high-performance liquid chromatography (HPLC) with the Surveyor liquid chromatography system (Thermo Scientific, USA) was used. The following parameters of the process were used: Aminex HPX-87H column (300 × 7.8 mm), refractometric RI and UV detector, 0.005 M L^−1^ sulphuric acid as eluent, flow rate 0.6 μL min^−1^, single sample analysis time 40 min.

### Statistical analysis

The normality of the distribution of variables was tested with Shapiro-Wilk’s test, and the homogeneity of variances was examined with Bartlett’s test^27^. Following the confirmation of normality and equal variance, results were analyzed with a one-way ANOVA test and Tukey’s post hoc test. Differences between samples with normal distribution were also evaluated by Student’s t-test. Furthermore, Principal components’ analysis (PCA) of overall diversity intestinal microbiota, parameters of chicken performance and the profile of fatty acids in the excreta of chickens were performed to compare all groups of animals at the time of treatment. Statistical analysis was performed using XLSTAT Software (Addinsoft, SARL, Paris, France) at the significance level of P < 0.05^27^. The results were presented as mean ± standard deviation (SD).

### Ethical approval and informed consent

Experiments were conducted after obtaining the approval of Local Ethical Commission No. 3 for animal testing at SGGW according to resolution No. 3/2015 (from 22 January 2015). All experiments were performed in accordance with the appropriate guidelines and regulations.

## Results

### The effect of probiotics and synbiotics on chicken’s performance

The basic parameter examined in terms of the effectiveness of probiotics, prebiotics or synbiotics in chickens is the assessment of the effect of preparations on performance of animals. During the experiment, all animals showed no adverse effect of being fed with the feed supplemented with the newly elaborated synbiotic preparations. In addition, no undesirable clinical symptoms (diarrhoea, constipation) nor post-mortem changes (inflammatory changes in the gut or other) were found.

The average body weight of chickens in the tested groups was significantly different in groups with synbiotic A, B, C and Cylactin (110.7 g, 107.5 g, 108.3 g and 120.7 g respectively) in comparison to the control group (137.9 g) at the age of seven days (Table S2). The body weight statistical differences were also noted at the age of 28 and 42 days of bird’s life. Chickens given feed with synbiotic A, B, C and Cylactin had lower average body weight (1944 g, 2095.3 g, 2096.2 g and 2119.6 g respectively) in comparison with the control group (2235 g) after 42 days.

Similar changes were noted in regard to the EPEF parameter. It was significantly different at day seven of bird life in groups fed with synbiotic A, B, C and Cylactin and at age 42 in the group of chickens fed with synbiotic A in comparison to the control group. EPEF ranged from 175.3 (synbiotic C) to 198.2 (Cylactin) when in the control group was 232.4 at day seven. Moreover, EPEF in chickens fed with synbiotic A was also lower compared to control at 42 day. EPEF ranged in that period from 280.96 to 306.69 in experimental groups when this parameter calculated for control group was 309.89 (Table S2). Another zootechnical parameter measured in our study was FCR. We did not notice any difference between the examined groups and control at days 7 and 28. The parameter differed at day 14 in groups fed with synbiotic C and probiotic BioPlus YC (1.10) and synbiotics (A, B and C) at day 42 (1.63, 1.60, 1.61 respectively) in comparison with control at the corresponding days (1.19 and 1.70) (Table S2).

**Figure 2:**
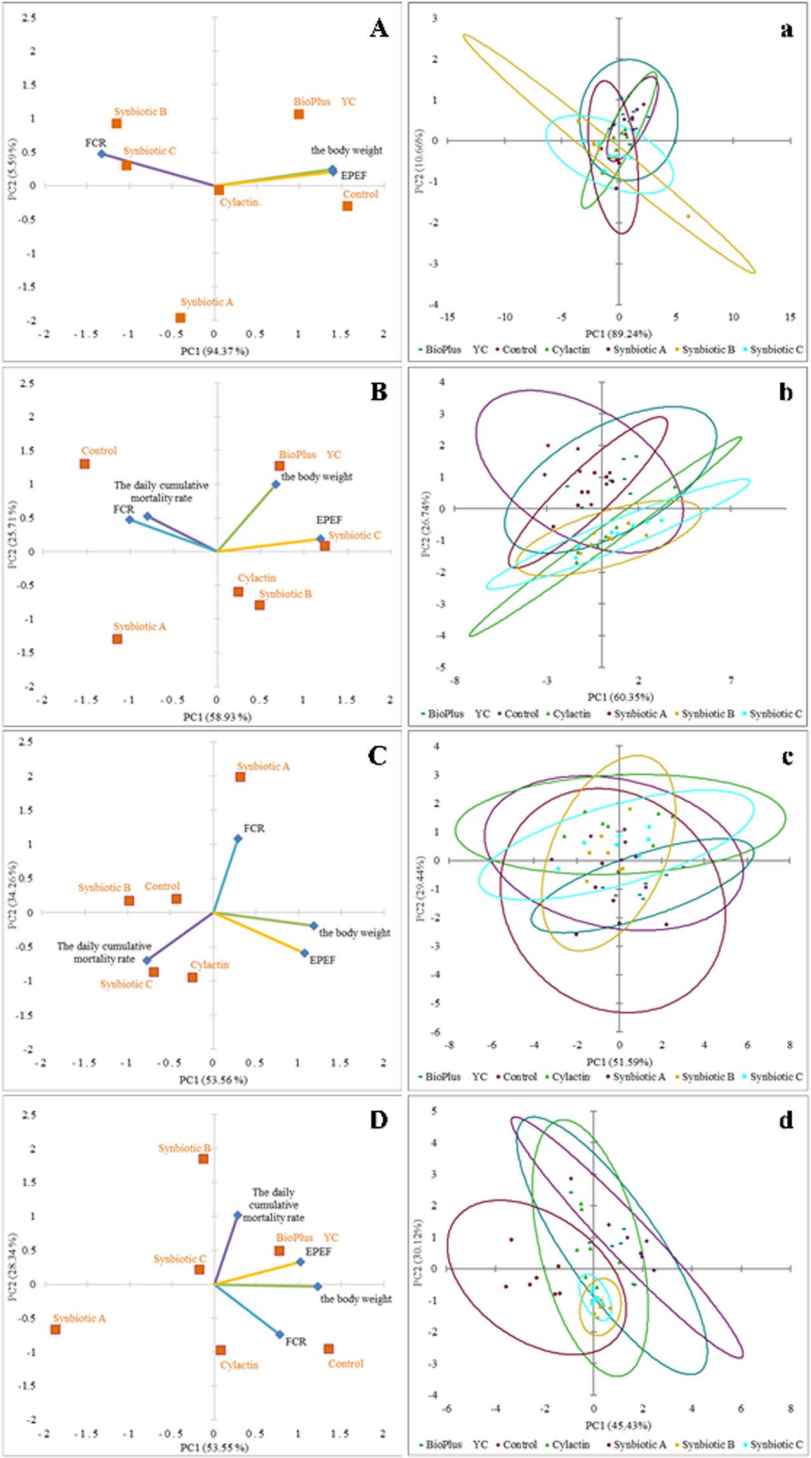
PCA plot of performance of chickens fed with the synbiotic or probiotic-supplemented feed and control group (without feed additives) after: (**A**) 7; (**B**) 14; (**C**) 28 and (**D**) 42 days of animals rearing; (**a**–**d**) Scatter plot showing a clustering of individuals in groups of animals in each time point of rearing respectively.

In addition, in order to better visualize the effect shown by the tested probiotics and synbiotics for chicken performance, principal component analysis (PCA) was used. The biplot for PC1 and PC2 showed the parameters with the greatest impact on the performance of broiler chickens in the tested groups after 7, 14, 28 and 42 days (Fig. 2A–D). The scatter plot visualization showed a distinct clustering of individuals in each tested group of animals (Fig. 2a–d).

### The effect of synbiotics on intestinal microbiota

The number of anaerobic bacteria in the content of the jejunum, the caecum and the excreta did not change significantly over the 42 days of chickens rearing. The total number of anaerobic bacteria was on average 1.13 × 10^6^ CFU g^−1^ (the jejunum), 2.37 × 10^7^ CFU g^−1^ (the caecum) and 4.66 × 10^6^ CFU g^−1^ (the excreta) on the second day of animal rearing. In a further period of rearing, no differences were statistically significant between the groups of animals and after 42 days, the number of anaerobic bacteria was at the level of 1.61 × 10^9^–7.10 × 10^9^ CFU g^−1^ (the jejunum), 7.22 × 10^9^–1.55 × 10^10^ CFU g^−1^ (the caecum) and 7.33 × 10^9^–1.53 × 10^10^ CFU g^−1^ (the excreta) (Tables S3–S5).

In samples of the intestinal content and the excreta, the mean count of *Enterobacteriaceae* family bacteria was similar in chickens fed with fodder supplemented with synbiotics, probiotics and the control group. After 42 days of chicken rearing, the total number of *Enterobacteriaceae* family bacteria was on average 1.69–9.03 × 10^7^ CFU g^−1^ (the jejunum), 1.61–5.67 × 10^8^ CFU g^−1^ (the caecum), 1.57–5.67 × 10^8^ CFU g^−1^ (the excreta) (Tables S3–S5).

Feeding chickens with feed supplemented with synbiotics resulted in a significant increase in the number of *Bifidobacterium* spp. and *Lactobacillus* spp. bacteria in the intestinal content and the excreta of animals, with the highest growth of these bacteria in the group of animals receiving synbiotic C. In the case of the newly elaborated synbiotic preparations, the average number of *Bifidobacterium* spp. bacteria were 1.47–6.43 × 10^9^ CFU g^−1^ (the jejunum), 4.90–8.73 × 10^9^ CFU g^−1^ (the caecum) and 3.70–9.13 × 10^9^ CFU g^−1^ (the excreta) after 42 days of rearing. In the content of intestines and the excreta, the number of *Bifidobacterium* spp. bacteria in chickens fed with feed supplemented with synbiotic C were respectively 2 and 1 order of magnitude higher compared to the control group in which the number of these bacteria increased slightly. Furthermore, the number of *Lactobacillus* spp. bacteria in the content of intestines and the excreta of chickens fed with fodder with synbiotic C was on average of 2–4 orders of magnitude higher than in control group on the 42^nd^ day of animal’s life. In the result, after 42 days of feed supplementation with synbiotic C, the count of *Lactobacillus* spp. bacteria in the caecum content was on average 1.01 × 10^9^ CFU g^−1^, while for synbiotic A and synbiotic B the counts were 1.62 × 10^8^ and 2.44 × 10^8^ CFU g^−1^ respectively. The tested probiotic preparations also had the beneficial effect on increase in the number of *Bifidobacterium* spp. and *Lactobacillus* spp. bacteria in the intestinal content and the excreta of broilers. However, significant statistical differences compared to the control group were found only in the jejunum in the case of *Lactobacillus* spp. after supplementation of feed with Cylactin (Tables S3–S5).

The count of *Clostridium* spp. and *Escherichia coli* bacteria did not change significantly in the jejunum and the caecum content of control chickens during breeding. However, the increase in the number of these microorganisms was found in excreta samples of the control group. In the intestinal content of chickens fed with symbiotic-supplemented fodder, the numbers of *Clostridium* spp. and *Escherichia coli* bacteria were significantly lower than control group after 7 (the jejunum) and 14 days (the caecum) respectively. The supplementation of the fodder for 42 days resulted in a decrease in the count of *Clostridium* spp. and *Escherichia coli* bacteria in the excreta and the tested parts of the intestine of chickens on average of 4 orders of magnitude compared to the control group. The best of result was found after the feed supplementation with synbiotic C. The reduction of the count of *Clostridium* spp. and *Escherichia coli* bacteria to the average on 2.63 × 10^4^ CFU g^−1^ and 7.73 × 10^3^ CFU g^−1^ respectively was found in the jejunum content of chickens. The decrease of the number of these bacteria was to the average of 4.00 × 10^5^–1.90 × 10^6^ CFU g^−1^ (synbiotic C and A), and 1.10–2.90 × 10^4^ CFU g^−1^ (synbiotic C and A) respectively after 42 days of animals breeding. In excreta samples of chickens fed with synbiotics, the reduction of the count *Escherichia coli* and *Clostridium* spp. bacteria to 3.00 × 10^5^ and 1.13 × 10^4^ CFU g^−1^ respectively was found. Furthermore, probiotic preparations (BioPlus YC and Cylactin) caused the reduction in the number these bacteria by the average of 1–2 orders of magnitude compared to control group (Tables S3–S5).

The administration of synbiotic or probiotic preparations to broiler chickens did not have a statistically significant effect on the number of *Enterococcus* spp. and *Bacteroides* spp. bacteria in the intestinal content and the excreta of the test animals (Tables S3–S5).

During the animals’ breeding, no statistically significant changes in number of yeast in chickens fed with probiotics or control animals were found. However, in the case of chickens fed with synbiotics as feed additives, a significantly higher count of yeast (the average of 3 orders the magnitude after 42 days) compared to the control group was found (Tables S3–S5).

**Figure 3:**
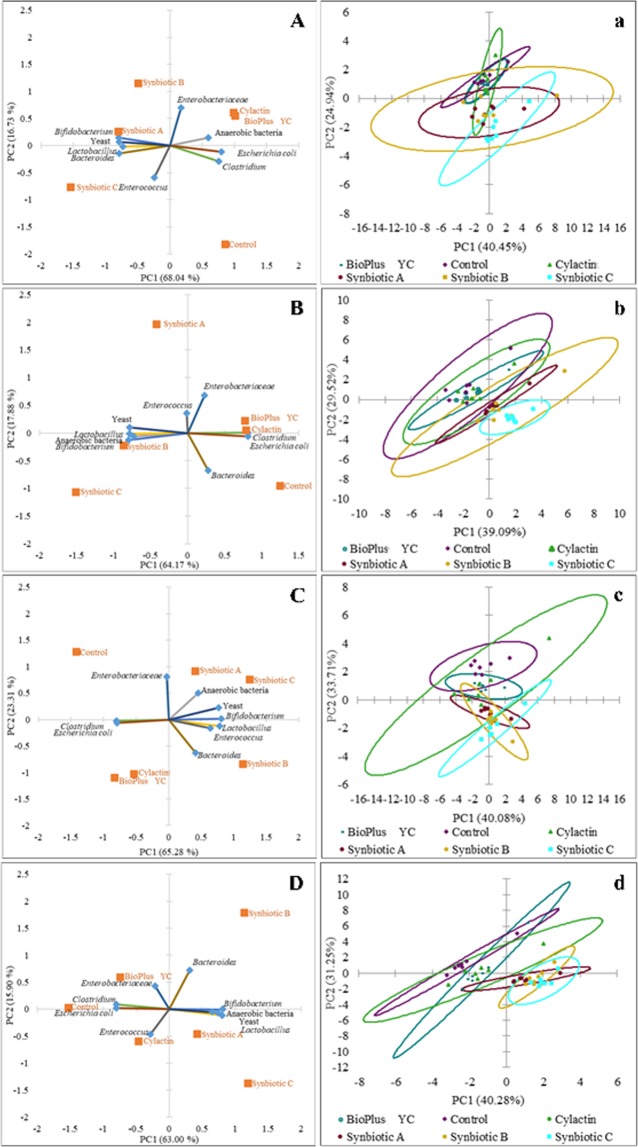
Principal component analysis (PCA) plot of counts of microorganisms which dominate in the jejunum content of chickens fed with the synbiotic or probiotic-supplemented feed and control group (without feed additives) after: (**A**) 7; (**B**) 14; (**C**) 28 and (**D**) 42 days of animals rearing; (**a**–**d**) Scatter plot showing a clustering of individuals in groups of animals in the each time point of rearing respectively.

**Figure 4:**
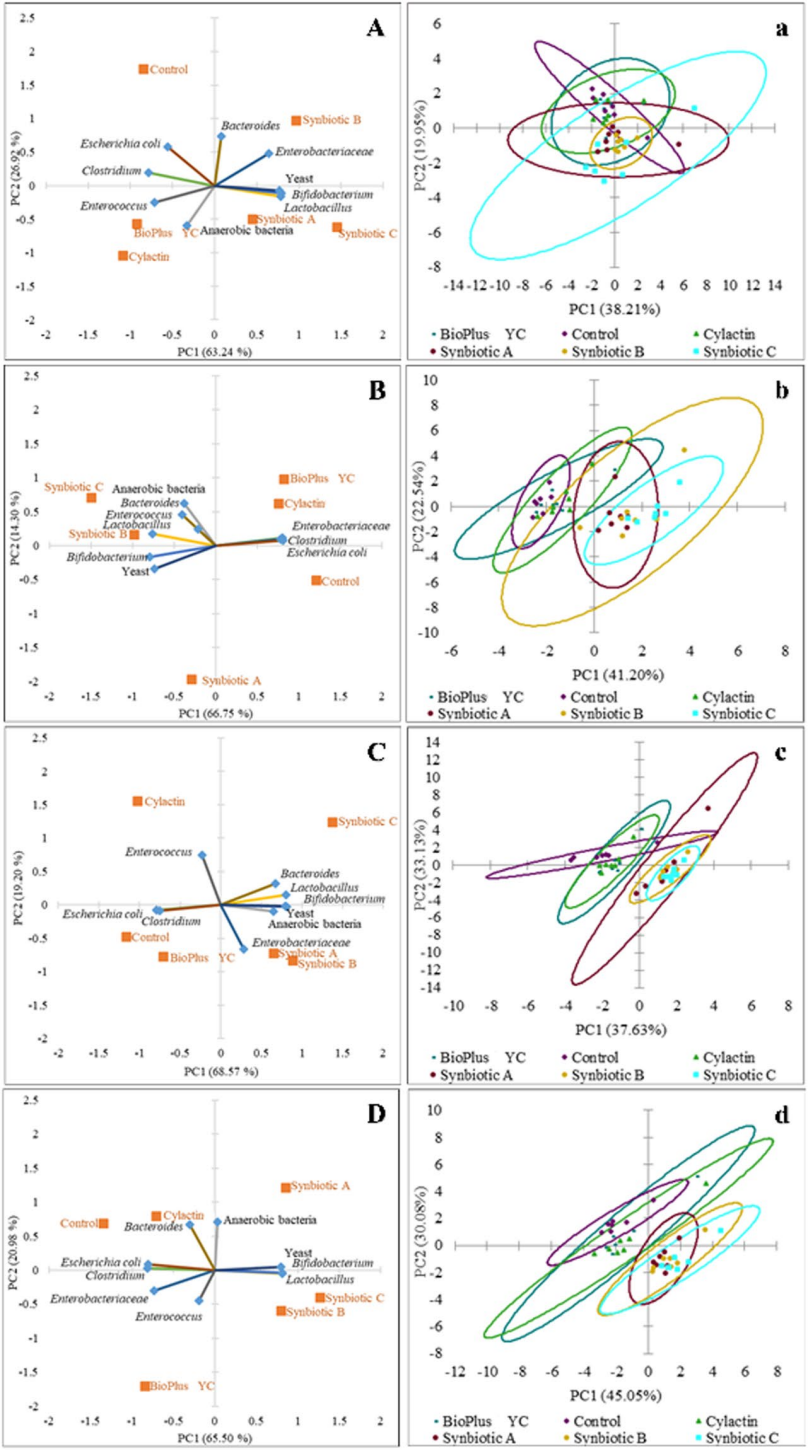
Principal component analysis (PCA) plot of counts of microorganisms which dominate in the caecum content of chickens fed with the synbiotic or probiotic-supplemented feed and control group (without feed additives) after: (**A**) 7; (**B**) 14; (**C**) 28 and (**D**) 42 days of animals rearing; (**a**–**d**) Scatter plot showing a clustering of individuals in groups of animals in the each time point of rearing respectively.

**Figure 5:**
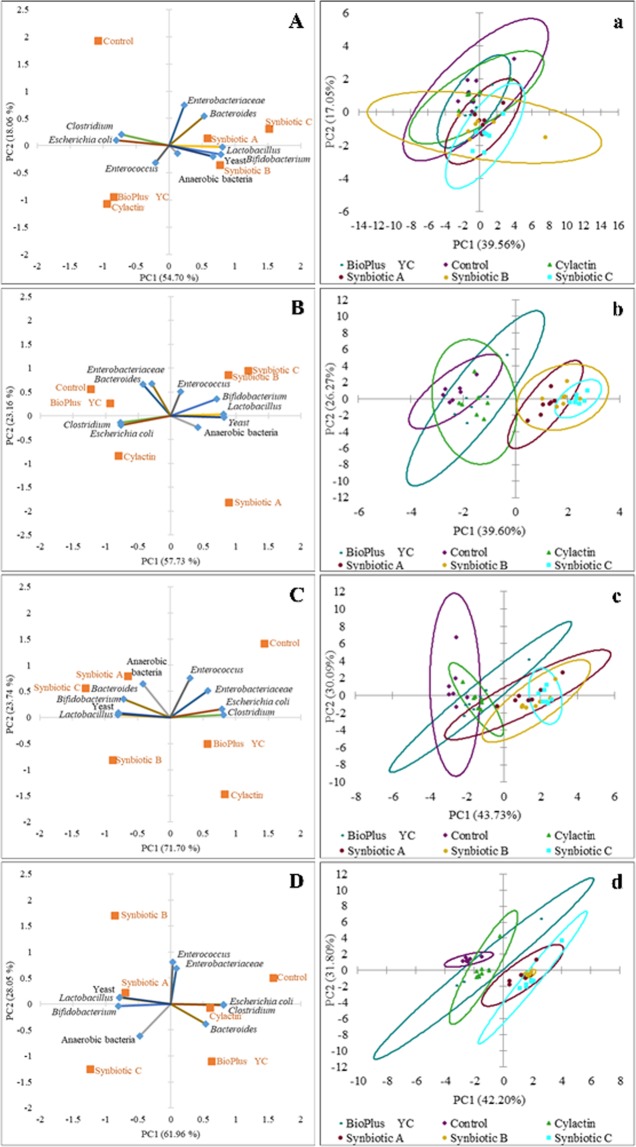
Principal component analysis (PCA) plot of counts of microorganisms which dominate in the excreta of chickens fed with the synbiotic or probiotic-supplemented feed and control group (without feed additives) after: (**A**) 7; (**B**) 14; (**C**) 28 and (**D**) 42 days of animals rearing; (**a**–**d**) Scatter plot showing a clustering of individuals in groups of animals in the each time point of rearing respectively.

In order to better visualize the results, PCA was used. The biplot for PC1 and PC2 showed the influence of tested formulas and commercial preparations on microbiota of the jejunum (Fig. 3), the caecum (Fig. 4) and the excreta (Fig. 5) in tested groups after 7, 14, 28 and 42 days (A, B, C and D respectively). The scatter plot visualization showed a distinct clustering of individuals in each tested group of animals (a, b, c and d in the Figs. 3–5).

### The profile of fatty acids in the excreta of chickens

The effect of synbiotic preparations on the metabolism of the intestinal microbiota was assessed. The type and proportions of microorganisms present in the intestine, or the so-called enterotype, may determine which products of the metabolism (both beneficial and harmful) are present in the host organism. SCFA are beneficial products. The study analyzed the lactic acid content and profiles of short-chain and branched-chain fatty acids as markers of animal welfare.

On the second day of life, the mean lactic acid level was 17.86 ± 1.26 μM g^−1^, and the mean total SCFA level was 8.42 ± 0.93 μM g^−1^. Mean levels of SCFA, such as acetic acid, propionic acid, butyric acid, valeric acid and formic acid, were respectively 5.74 ± 0.90 μM g^−1^, 0.46 ± 0.19 μM g^−1^, 0.49 ± 0.03 μM g^−1^, 0.20 ± 0.07 μM g^−1^, 1.53 ± 0.15 μM g^−1^. The total level of branched-chain fatty acids (BCFA) in chicken the excreta after the second day of rearing was 0.42 ± 0.16 μM g^−1^, including 0.30 ± 0.11 μM g^−1^ of isobutyric acid, and 0.12 ± 0.06 μM g^−1^ of isovaleric acid (Table S6).

The supplementation with synbiotic preparations carried out for 42 days caused a significant increase in the level of lactic acid in the excreta of chickens, ranging between 22.61 ± 2.35 and 27.32 ± 3.66 μM g^−1^ for synbiotic A and C respectively. The sum of SCFA concentration increased significantly in groups of chickens fed of feed with synbiotics and was from 18.99 ± 2.99 to 20.65 ± 1.89 μM g^−1^ for synbiotic A and C respectively, wherein the least increase of concentration was observed in the case of valeric acid. However, a significant increase of the level after 42 days of administration of synbiotic preparations with feed was observed in the case of acetic, propionic, butyric and formic acid, which were 13.33 ± 1.08–13.81 ± 1.98 μM g^−1^, 0.89 ± 0.22–0.97 ± 0.21 μM g^−1^, 1.89 ± 0.31–2.48 ± 0.96 μM g^−1^ and 2.43 ± 0.23–2.99 ± 0.34 μM g^−1^ respectively. In the control group, the lactic acid and SCFA concentration after 42 days of rearing were maintained on a similar level as that measured after two days of rearing and were 17.90 ± 1.26 μM g^−1^ and 9.04 ± 0.60 μM g^−1^ respectively (Table S5). At the same time, a significant decrease of BCFA (isobutyric and isovaleric acids) levels was observed in the excreta of chickens fed with synbiotic-supplemented feed. Concentrations of those acids were 0.12 ± 0.07–0.15 ± 0.09 μM g^−1^ and 0.06 ± 0.02–0.07 ± 0.04 μM g^−1^ respectively after 42 days of rearing (Table S6).

Probiotic preparations (BioPlus YC and Cylactin) also had a beneficial effect on the level of lactic acid, SCFA and BCFA produced by the intestinal microbiota of chickens. However, the observed differences in concentrations of these acids were not as statistically significant as in the case of the elaborated synbiotic preparations. The most beneficial effect on fatty acids profile in the excreta of chickens after 42 days of administration with feed was observed in the case of synbiotic C (Table S6).

**Figure 6:**
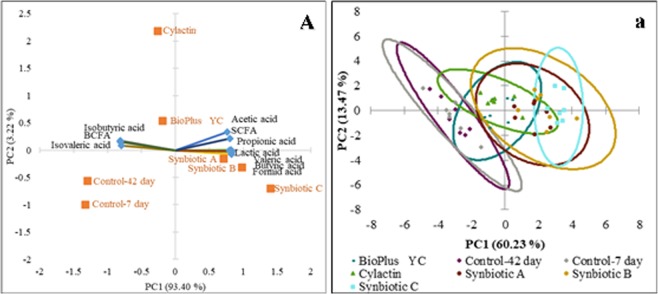
(**A**) Principal component analysis (PCA) plot of lactic acid, SCFA and BCFA concentrations in the excreta of chickens after 7 and 42 days. This figure shows compounds responsible for divergence between groups of animals; (**a**) Scatter plot showing a clustering of individuals in groups of animals sfter 7 and 42 days of rearing respectively.

Moreover, in order to better visualize the results, PCA was used. The biplot for PC1 and PC2 showed the influence of tested formulas and commercial preparations on individual acids in the excreta in tested groups after 7 and 42 days (Fig. 6A). The visualization with use the scatter plot showed a distinct clustering of individuals in each tested group of animals (Fig. 6a).

## Discussion

The aim of our work was to investigate of the influence of newly elaborated synbiotic preparations, used as addition to feed, on the count of dominant intestinal microorganisms in chickens, and on the profile of fatty acids (SCFA, BCFA) and lactic acid produced. Moreover, we also tested parameters of the performance of animals such as daily cumulative mortality rate, feed conversion ratio (FCR) and European Production Efficiency Factor (EPEF).

The performance of chickens is often used for the evaluation of the influence of synbiotics (probiotics/prebiotics) on broiler chickens health. In many experimental models, it has been shown that these nutritional supplements usually improve the performance of broiler chickens, but the results depend on type of synbiotics used^37–47^. Usually parameters of the performance are measured at the end of the production cycle, but for improved analysis we decided to check it at each time point.

The starting point of the research was determination of chicken performance. We observed that the body weight of chickens was lower in the group fed with synbiotics and Cylactin on day 7 and 42, in comparison to the control group. However, the FCR was lower in groups A, B and C on day 42. This parameter clearly indicates that birds fed with synbiotics consume less feed per 1 kg of body weight gain than birds from control group, which is positive and desirable from an economic point of view. The differences in body weight may be due to inequality between sexes in different groups. We chose birds for testing randomly from the groups, which is why FCR seems to better reflect the reality parameter. Moreover, we noticed this value was lower in all groups receiving probiotics, which is a sign of better production efficiency and may confirm the positive influence of probiotics on chicken breading. The performance of broilers was also evaluated in terms of the European Production Efficiency Factor (EPEF), which includes daily weight gain and survival percentage. Higher values of EPEF indicate that the birds’ body weight gain is uniform, and the flock is in good health^48^. The EPEF value in chickens fed with feed with synbiotic A at 42 days of age was significantly lower, but there were no statistically significant differences between results from other groups in comparison to the control group. Based on the obtained results, it was found that the newly elaborated synbiotic preparations (A, B and C) had a beneficial effect on parameters the performance of chickens such as daily cumulative mortality rate (%), feed conversion ratio (FCR), and the European Production Efficiency Factor (EPEF).

The next step was determination of the dominant microorganisms in intestinal content (the jejunum and the caecum) and in the excreta of tested chickens. The composition of the gut microbiota of broiler chickens is affected by multiple factors and the count of dominant microorganisms change from the first day of animal life. The number of microorganisms in sections of the digestive system can vary significantly^49^. On the first day, *coli* group bacteria, enterococci and lactic bacteria predominate in the chicken goiter, duodenum and small intestine^50,51^. Their initial count is low, but already after 5–6 hours it ranges between 10^9^ and 10^10^ CFU g^−1^ of the excreta. After the first week of broiler life, *Lactobacillus* genus bacteria predominate, and they also colonize the goiter epithelium. In the second week of chicken life, lactic bacteria predominate in the small intestine and the duodenum. Starting from the third week of life, the ileum microbiota consists of *Lactobacillus* spp. (70%), *Clostridium* spp. (11%), *Streptococcus* spp. (6.5%), *Enterobacteriaceae* family bacteria (6.5%), *Enterococcus* spp. (6%)^52^. The microbiota of the caecum is like that of the small intestine on the first day of life. However, it is significantly changed in 30-day old chickens. *Bacteroides* spp., *Eubacterium* spp., *Clostridium* spp. and *Ruminococcus* spp. become dominant in this time^53^.

In all tested groups of chickens, the total anaerobic bacteria count in the intestinal content and the excreta were comparable and the mean value was 10^9^–10^10^ CFU g^−1^ after 42 days. Therefore, it has been found that feeding chickens with synbiotics (A, B and C) and commercial probiotics (Cylactin and BioPlus YC) for 42 days caused changes in the count of specific groups of microorganisms but did not significantly affect the total count of anaerobic bacteria. This is a beneficial effect, as the balance of intestinal microbiota in chickens is maintained. Our results on total anaerobic bacteria count were higher, on average by one to two orders of magnitude, compared to the results reported by Dibaji, Seidavi, Asadpour, & da Silva, who tested the effect of the Biomin IMBO synbiotic, containing the probiotic bacteria *Enterococcus faecium* (5 × 10^11^ CFU kg^−1^) and fructooligosaccharides (a prebiotic), administered for 42 days, on the microbiota of chicken caecum^54^. In addition, the mean count of *Enterobacteriaceae* family bacteria was similar in each group of chickens. After 42 days of chicken rearing, the total number of *Enterobacteriaceae* family bacteria was on average 10^7^ CFU g^−1^ (the jejunum) and 10^8^ CFU g^−1^ (the caecum and the excreta). Some comparable results were obtained in the study by Biernasiak, Śliżewska, Libudzisz, & Smulikowska, when the *Enterobacteriaceae* family bacteria count in the excreta of chickens receiving probiotics was 10^8^ CFU g^−1^ ^55^. The administration of the elaborated synbiotics and commercial probiotics to broiler chickens did not have a statistically significant effect on the number of *Enterococcus* spp. and *Bacteroides* spp. bacteria in the intestinal content and the excreta of animals. Lan, Binh, & Banno evaluated the effect of two probiotics on the *Enterococcus* spp. count in droppings of chickens also did not find any significant differences compared to the control group^56^. There are no literature data presenting the results of studies using the culture method, regarding the effect of probiotic, prebiotic or synbiotic supplementation on the count of *Bacteroides* spp. in the gastrointestinal tract of broilers. Therefore, our study is completely original in this regard. The beneficial effect of synbiotics (A, B and C) supplementation has been found also due to a significant increase in the number of *Bifidobacterium* spp. and *Lactobacillus* spp. bacteria in the intestinal content and the excreta of animals. The highest growth of these bacteria was in the group of animals receiving the most complex preparation (synbiotic C). The average number of *Bifidobacterium* spp. and *Lactobacillus* spp. bacteria in chickens fed with feed supplemented tested synbiotics was 10^9^ CFU g^−1^ and 10^8^–10^9^ CFU g^−1^ respectively. On the other hand, the addition of synbiotics to chicken feed caused a significant reduction in potentially pathogenic bacteria such as *Clostridium* spp. and *Escherichia coli*. The supplementation of the fodder for 42 days resulted in a decrease of these bacteria in the content of the intestine and the excreta of chickens on average of four orders of magnitude compared to the control group. Again, the best result was found after feed supplementation with the synbiotic C. Furthermore, the probiotic preparations (BioPlus YC and Cylactin) caused the reduction in the number these bacteria by the average of 1–2 orders of magnitude compared to control group. Yeast is not a natural intestinal microbiota of chickens. Only in the group of broilers fed with the elaborated synbiotics as feed additives, was a significantly higher total count of yeast (the average of three orders the magnitude after 42 days) found compared to the control group. These results are very promising, because synbiotic preparations administered to chickens contained *Saccharomyces cerevisiae*. Therefore, a high total count of yeast in samples obtained from animals fed with the synbiotic-supplemented diet may indicate the survival of those microorganisms in the gastrointestinal system.

In the last stage of our research, the effect of synbiotic preparations on the metabolism of the intestinal microbiota was assessed. The lactic acid content and profiles of short-chain and branched-chain fatty acids in the excreta of broiler chickens was determined.

The supplementation with synbiotic preparations carried out for 42 days caused a significant increase of the level of lactic acid and the sum of SCFA (acetic, propionic, valeric, butyric and formic acids) in the excreta of chickens compared to the control group. At the same time, a significant decrease of BCFA (isobutyric and isovaleric acids) levels was observed in the excreta of chickens fed with synbiotic-supplemented feed. Commercial probiotic preparations (BioPlus YC and Cylactin) also had a beneficial effect on the level of lactic acid, SCFA and BCFA produced by the intestinal microbiota of chickens but differences in concentrations of these acids were not as statistically significant as in the case of the elaborated synbiotic preparations. The most beneficial effect on fatty acids profile in the excreta of chickens was observed in the case of synbiotic C.

SCFA play a very important role in the regulation of pH, increased calcium, iron and magnesium absorption, and also have a beneficial effect on the hepatic metabolism of glucose and proteins. There acids are very important in the maintenance of the proper structure, function and integrity of the intestine. Moreover, by stimulating the growth of saprophytic flora, SCFA inhibit the growth of potentially pathogenic microorganisms, such as *Salmonella*, *Escherichia coli* and *Campylobacter*, as they compete for the colonization site^57^. On the other hand, excessive accumulation of SCFA (isobutyric and isovaleric acids) suggests an abnormal course of fermentation and digestion. They are putrid acids and their increased production may be associated with an excessively high amount of non-absorbed amino acids or proteins reaching the caecum. BCFA are metabolized by enterocytes, i.e. cells of the small intestine. Their high concentration may also indicate the presence of blood in the intestinal content, or too intense a growth of pathogenic microbiota in the small intestine, where proteins are readily available^58^. The profile of fatty acids changes under the influence of numerous factors such as diet, external environment, health and the intestinal microbiota of animals. Meimandipour *et al*. demonstrated variable levels of SCFA and lactic acid in the gastrointestinal tract of chickens at various stages of development. The highest accumulation of those metabolites was found in the caecum of 14-day old broiler chickens (in the following ratio: lactic acid: acetic acid: propionic acid: butyric acid - 49:37:11:3)^59^. After 28 days the proportion was 12:73:6:10, and the highest level of lactic acid was found in each section of the small intestine^59^. The analysis of the extrema conducted in our study demonstrated a similar relationship, i.e. the highest share of lactic acid in the total amount of determined acids.

In summary, our results showed a beneficial effect of all elaborated synbiotics on the balance of intestinal microbiota, their metabolism and the performance of broiler chickens. It was found that new synbiotics were effective in improving parameters the performance of chickens. All synbiotic formulas caused increase in the number of beneficial intestinal microorganisms (*Bifidobacterium* spp. and *Lactobacillus* spp.) and a significant reduction in potentially pathogenic bacteria such as *Clostridium* spp. and *Escherichia coli* in the intestinal contents and the excreta of animals. Moreover, synbiotics resulted beneficial changes in the metabolism of the intestinal microbiome of chickens, causing a significant increase of the level of lactic acid and the sum of SCFA with significant decrease of BCFA in the excreta of animals compared to the control group. New elaborated synbiotic preparations had a more beneficial effect on chicken health than tested probiotics. Synbiotic C (the most complex preparation) proved to be the most effective one. Hence, elaborated synbiotics can be successfully used as feed additives for broiler chickens. Further studies are needed to explain the mechanisms of the observed influence of the synbiotics on broilers health.

## Supplementary information

Supplementary information

## Data Availability

The data analysed are publicly available in source articles and data citations were included in the reference list.
