# Supplementary material for: The effect of synbiotic preparations on the intestinal microbiota and her metabolism in broiler chickens
Source: Sci Rep. 2020 Mar 9;10:4281. doi: 10.1038/s41598-020-61256-z (PMC7062770; doi:10.1038/s41598-020-61256-z)
Supplement: Supplementary file 1 — Supplementary information [file 41598_2020_61256_MOESM1_ESM.docx]

**The effect of synbiotic preparations** **on the intestinal microbiota and her metabolism in broiler chickens**

Katarzyna Śliżewska^1*^, Paulina Markowiak-Kopeć^1*^, Artur Żbikowski^2^, Piotr Szeleszczuk^2^

^1^ Institute of Fermentation Technology and Microbiology, Faculty of Biotechnology and Food Sciences, Lodz University of Technology, Wolczanska 171/173, 90-924 Lodz, Poland

^2^ Department of Pathology and Veterinary Diagnostics, Faculty of Veterinary Medicine, Warsaw University of Life Sciences – SGGW, Nowoursynowska 159c St., 02-776 Warsaw, Poland

* Corresponding authors: [katarzyna.slizewska@p.lodz.pl](mailto:katarzyna.slizewska@p.lodz.pl), paulina.markowiak@edu.p.lodz.pl

**SUPPORTING INFORMATION**

Supporting information be found in the online version of this article:

**Table S1.** The composition of the complete dietetic commercial mixtures of feed applied in the experiments *^28^*.

|  | Type of feed | | |
| --- | --- | --- | --- |
| Name | **Broiler Starter Prestige** | **Broiler Grower Prestige** | **Broiler Finisher Prestige** |
| Composition | wheat, corn, post-extraction soya meal*, haemoglobin (from swine blood), rapeseed cake, soybean oil*, calcium carbonate, palm oil derived fatty acids, monocalcium phosphate, sodium chloride | wheat, corn, post-extraction soya meal*, haemoglobin (from swine blood), rapeseed cake, post-extraction sunflower meal, palm oil derived fatty acids, soybean oil*, calcium carbonate, swine fat, monocalcium phosphate, sodium chloride | wheat, corn, post-extraction soya meal*rapeseed cake, soybean oil*, calcium carbonate, monocalcium phosphate, sodium chloride |
| Component amount in 1 kg of feed | | | |
| Total proteins (Kjeldahl method) | 225 g | 205 g | 187.5 g |
| Oil and crude fats | 48 g | 55 g | 75 g |
| Fibre | 27 g | 30 g | 33 g |
| Lysine | 13.6 g | 12.9 g | 12.2 g |
| Methionine | 6 g | 5.8 g | 5.6 g |
| Calcium | 8 g | 6.2 g | 4.9 g |
| Phosphorus | 6 g | 5.2 g | 4.6 g |
| Sodium | 1.5 g | 1.5 g | 1.4 g |
| Ash | 55 g | 47 g | 40 g |
| Dietetic additives in 1 kg of feed | | | |
| Vitamin A (3a672a) | 10000 u.m. | | |
| Vitamin D3 (E671) | 5000 u.m. | | |
| Vitamin E (dl-α-tocopherol) | 75 mg | | |
| Fe (iron sulphate, E1) | 40 mg | | |
| J (potassium iodide, 3b201) | 1.25 mg | | |
| Cu (copper sulphate, E4) | 16 mg | | |
| Mn (manganese oxide, E5) | 120 mg | | |
| Zn (zinc oxide, E6) | 100 mg | | |
| Se (sodium selenite, E8) | 0.3 mg | | |
| Zootechnics additives in 1 kg of feed | | | |
| 6-phytase (EC 3.1.3.26) 500 FTU g^-1^, 4a19 | 1000 FTU | 1000 FTU | - |
| 6-phytase (EC 3.1.26) 2500 OTU g^-1^, 4a16 | - | - | 250 OTU g^-1^ |
| Endo-1,4-β-xylanase (EC 3.2.1.8),  12500 VU ml^-1^, 4a22 | 1250 VU | 1250 VU | - |
| Endo-1,4-β-xylanase (EC 3.2.1.8), 30000 EPU g^-1^, 4a1617 | - | - | 1500 EPU |
| Endo- 1,3 (4)-β-gluconate (EC 3.2.1.6), 8600 VU ml^-1^, 4a22 | 860 VU | 860 VU | - |
| Serine protease (EC 3.4.21), 75000 PROT g^-1^ 4a13 | 15000 PROT | - | - |

**EPU** - one endo-1,4-β-xylanase unit is the amount of enzyme which releases 0.0083 μM of reducing sugars (xylose equivalent) per minute from oat spelt xylan at pH 4.7 and 50°C; **FTU** - one 6-phytase unit is the amount of enzyme which liberates 1 µM of inorganic phosphate from sodium phytate in one minute at 37°C and pH 5.5; **PROT** - one protease unit is the amount of enzyme that releases 1 µM of p-nitroaniline from 1mM substrate (Suc-Ala-AlaPro-Phe-pNA) per minute at pH 9.0 and temperature 37 ºC**; VU** – one endo-1,3(4)-β-gluconate unit is the amount of enzyme which hydrolyzes substrate (β-glucan of barley and arabinoxylan of wheat respectively), while reducing viscosity of the solution, so that there has been a change in relative fluidity of 1 (dimensionless unit) in one minute at 30°C and pH 5,5; *** -** produced from GMO soybeans (MON 40-3-2).

**Table S2.** Rearing parameters of chickens fed with the synbiotic or probiotic-supplemented feed after 42 days of life. Results significantly different from: ^*^ the control group; group of chickens fed with: ^A^ Synbiotic A; ^B^ Synbiotic B, ^C^ Synbiotic C; ^b^ BioPlus YC; ^c^ Cylactin; one-way ANOVA with post-hoc Tukey’s test (P < 0.05).

| The age of birds (days) | Feed additives | The body weight  (mean±SD) (g) | Daily cumulative mortality rate (%) | FCR | EPEF |
| --- | --- | --- | --- | --- | --- |
| 7 | Synbiotic A | 110.71±12.02 ^b, c, *^ | 0 | 0.87±0.03 | 182.66±23.29 ^b, *^ |
|  | Synbiotic B | 107.53±11.82 ^b, c, *^ | 0 | 0.89±0.10 | 175.99±39.36 ^b, c, *^ |
|  | Synbiotic C | 108.32±7.81 ^b, c, *^ | 0 | 0.89±0.05 | 175.33±23.22 ^b, c, *^ |
|  | BioPlus YC | 134.43±7.53 ^A, B, C, c^ | 0 | 0.86±0.05 | 223.21±22.03 ^A, B, C, c^ |
|  | Cylactin | 120.66±10.35 ^A, B, C, b, *^ | 0 | 0.87±0.03 | 198.23±20.97 ^B, C, b, *^ |
|  | Without (control) | 137.93±8.48 ^A, B, C, c^ | 0 | 0.85±0.02 | 232.41±18.04 ^A, B, C, c^ |
| 14 | Synbiotic A | 288.65±23.01 | 1.19 ^B, C, c^ | 1.12±0.02 | 182.19±16.27 |
|  | Synbiotic B | 298.69±17.19 | 0 ^A, b, *^ | 1.11±0.05 | 193.01±17.77 |
|  | Synbiotic C | 305.60±26.73 | 0 ^A, b, *^ | 1.10±0.05 ^*^ | 199.86±25.85 |
|  | BioPlus YC | 308.42±23.60 | 1.19 ^B, C, c^ | 1.10±0.04 ^*^ | 198.85±20.55 |
|  | Cylactin | 299.80±38.32 | 0 ^A, b, *^ | 1.12±0.02 | 189.71±27.80 |
|  | Without (control) | 302.90±17.04 | 1.19 ^B, C, c^ | 1.19±0.08 ^C, b^ | 179.94±16.44 |
| 28 | Synbiotic A | 1121.33±130.45 | 0 ^B, C, c, *^ | 1.43±0.07 | 279.78±28.53 |
|  | Synbiotic B | 1087.50±64.17 ^b^ | 1.19 ^A, b, c^ | 1.39±0.06 | 276.87±17.13 ^b^ |
|  | Synbiotic C | 1096.33±116.77 | 1.19 ^A, b, c^ | 1.36±0.04 | 284.23±35.70 |
|  | BioPlus YC | 1180.17±79.61 ^B^ | 0 ^B, C, c, *^ | 1.39±0.04 | 307.75±26.60 ^B^ |
|  | Cylactin | 1130.00±122.76 | 2.38 ^A, B, C, b, *^ | 1.39±0.05 | 290.06±37.82 |
|  | Without (control) | 1113.67±134.02 | 1.19 ^A, b^ | 1.39±0.05 | 280.04±29.93 |
| 42 | Synbiotic A | 1944.00±66.28 ^B, C, b, c, *^ | 1.19 ^B, b^ | 1.63±0.03 ^B, b, *^ | 280.96±11.52 ^B, C, b, c, *^ |
|  | Synbiotic B | 2095.33±18.14 ^A, b, *^ | 2.38 ^A, C, c, *^ | 1.60±0.01 ^A, b, c, *^ | 304.43±2.52 ^A^ |
|  | Synbiotic C | 2096.17±12.43 ^A, b, *^ | 1.19 ^B, b^ | 1.61±0.01 ^b, c, *^ | 306.70±2.37 ^A, c^ |
|  | BioPlus YC | 2176.50±56.95 ^A, B^ | 2.38 ^A, C, c, *^ | 1.69±0.05 ^A, B, C^ | 299.62±14.79 ^A^ |
|  | Cylactin | 2119.67±39.51 ^A, *^ | 1.19 ^B, b^ | 1.68±0.05 ^B, C^ | 297.31±9.48 ^A, C^ |
|  | Without (control) | 2235.00±67.71 ^A, B, C, c^ | 1.19 ^B, b^ | 1.70±0.04 ^A, B, C^ | 309.90±16.67 ^A^ |

**Table S3.** Counts of microorganisms which dominate in the content of the jejunum of chickens fed with the synbiotic or probiotic-supplemented feed. The mean results from three repetitions for seven individuals from each group ± standard deviation (SD). Results significantly different from: ^*^ the control group; group of chickens fed with: ^A^ Synbiotic A; ^B^ Synbiotic B, ^C^ Synbiotic C; ^b^ BioPlus YC; ^c^ Cylactin; one-way ANOVA with post-hoc Tukey’s test (P < 0.05).

| Microorganism | Feed additives | | | | | |
| --- | --- | --- | --- | --- | --- | --- |
|  | **Synbiotic A** | **Synbiotic B** | **Synbiotic C** | **BioPlus YC** | **Cylactin** | **Without (Control)** |
|  | **log CFU g^-1^ (mean±SD)** | | | | | |
| Day 2 | | | | | | |
| Anaerobic bacteria | 6.05±0.24 | | | | | |
| *Lactobacillus* | 4.22±0.04 | | | | | |
| *Bifidobacterium* | 4.12±0.14 | | | | | |
| *Clostridium* | 4.37±0.13 | | | | | |
| *Enterobacteriaceae* | 5.48±0.30 | | | | | |
| *Escherichia coli* | 3.52±0.43 | | | | | |
| *Enterococcus* | 4.43±0.14 | | | | | |
| *Bacteroides* | 4.12±0.14 | | | | | |
| Yeast | 3.38±0.07 | | | | | |
| Day 7 | | | | | | |
| Anaerobic bacteria | 9.11±0.28 | 9.20±0.32 | 9.28±0.25 | 9.57±0.34 | 9.64±0.54 | 9.30±0.27 |
| *Lactobacillus* | 6.20±0.06 ^C, c^ | 6.31±0.08 ^C^ | 7.27±0.15 ^A, B, b, c, *^ | 5.81±0.45 ^C^ | 5.90±0.76 ^A, C^ | 5.65±0.26 ^C^ |
| *Bifidobacterium* | 7.30±0.27 ^C, *^ | 7.37±0.30 | 7.59±0.13 ^b, c, *^ | 6.34±0.46 ^C^ | 6.31±0.61 ^A, C^ | 6.27±0.09 ^A, C^ |
| *Clostridium* | 7.44±0.39^*^ | 7.32±0.27 ^*^ | 7.19±0.03 ^*^ | 8.10±0.46 | 8.16±0.43 | 8.49±0.18 ^A, B, C^ |
| *Enterobacteriaceae* | 7.48±0.04 | 7.82±0.19 | 7.45±0.32 | 7.63±0.61 | 7.70±0.55 | 7.41±0.16 |
| *Escherichia coli* | 6.22±0.03 ^b, c^ | 6.48±0.21 ^b, c^ | 6.10±0.31 ^b, c^ | 7.18±0.57 ^A, B, C^ | 7.09±0.67 ^A, B, C^ | 7.25±0.31 ^A, B, C^ |
| *Enterococcus* | 8.23±0.30 | 8.58±0.40 | 8.94±0.23 | 8.26±0.51 | 8.41±0.48 | 8.88±0.23 |
| *Bacteroides* | 7.59±0.37 | 7.37±0.42 | 7.67±0.14 | 7.11±0.67 | 7.12±0.71 | 7.25±0.21 |
| Yeast | 4.54±0.41 | 4.31±0.36 | 5.05±0.41 | 3.78±0.41^C^ | 3,88±0.66 ^C^ | 3.59±0.36 ^C^ |
| Day 14 | | | | | | |
| Anaerobic bacteria | 9.50±0.62 | 9.63±0.38 | 9.61±0.48 | 9.46±0.38 | 9.32±0.53 | 9.37±0.59 |
| *Lactobacillus* | 7.22±0.72 ^c, b, *^ | 7.38±0.72 ^c, b, *^ | 8.52±0.56 ^A, B, b, c, *^ | 6.30±0.47 ^A, B, C^ | 6.60±0.40 ^C, *^ | 5.54±0.43 ^A, B, C, c^ |
| *Bifidobacterium* | 7.73±0.43 | 8.03±0.56 | 8.27±0.53^b^ | 7.44±0.44 ^C^ | 7.54±0.32 | 7.31±0.34 |
| *Clostridium* | 6.75±0.49 ^C, c, *^ | 6.23±0.69 ^C, b, c, *^ | 5.72±0.67 ^A, B, b, c, *^ | 7.98±0.43 ^B, C^ | 7.97±0.40 ^A, B, C^ | 8.31±0.71 ^A, B, C^ |
| *Enterobacteriaceae* | 7.99±0.50 | 7.53±0.42 | 7.32±0.54 | 7.81±0.64 | 7.45±0.43 | 7.60±0.41 |
| *Escherichia coli* | 5.60±0.41 ^b^ | 5.45±0.39^b^ | 4.81±0.40 ^b, c^ | 7.05±0.29 ^A, B, C^ | 7.03±0.44 ^C^ | 7.34±0.36 |
| *Enterococcus* | 8.56±0.40 | 8.78±0.43 | 8.18±0.47 | 8.33±0.28 | 8.67±0.31 | 8.31±0.37 |
| *Bacteroides* | 7.85±0.42 | 8.08±0.41 | 8.00±0.52 | 8.02±0.53 | 7.98±0.51 | 8.16±0.44 |
| Yeast | 4.95±0.44 | 5.00±0.42 | 5.27±0.36 ^b, c, *^ | 3.68±0.28 ^C^ | 3.54±0.44 ^C^ | 3.65±0.57 ^C^ |
| Day 28 | | | | | | |
| Anaerobic bacteria | 9.73±0.43 | 9.79±0.47 | 9.79±0.50 | 9.48±0.52 | 9.61±0.30 | 9.76±0.47 |
| *Lactobacillus* | 8.26±0.75 ^C, b, c, *^ | 8.76±0.43 ^c, *^ | 9.12±0.50 ^A, b, c, *^ | 7.25±0.41 ^A, C, *^ | 7.16±0.43 ^A, B, C, *^ | 5.48±0.35 ^A, B, C, b, c^ |
| *Bifidobacterium* | 8.78±0.58 ^B, C, *^ | 9.59±0.26 ^A, b, c, *^ | 9.70±0.57 ^A, b, c, *^ | 8.09±0.39 ^B, C^ | 8.24±0.34 ^B, C^ | 7.80±0.43 ^A, B, C^ |
| *Clostridium* | 5.75±0.44 ^C, b, *^ | 5.34±0.65 ^C, b, *^ | 4.60±0.52 ^A, B, b, c, *^ | 7.44±0.30 ^A, B, C^ | 7.52±0.40 ^C^ | 8.80±0.45 ^A, B, C^ |
| *Enterobacteriaceae* | 7.87±0.45 | 7.17±0.32 | 7.74±0.42 | 7.24±0.32 | 7.13±0.43 | 7.88±0.55 |
| *Escherichia coli* | 4.49±0.43 ^b, *^ | 4.31±0.50 ^b, *^ | 4.01±0.42 ^b, *^ | 6.45±0.31 ^A, B, C^ | 6.73±0.43 | 7.63±0.50 ^A, B, C^ |
| *Enterococcus* | 8.73±0.47 | 8.85±0.48 | 8.95±0.40 | 8.64±0.46 | 8.87±0.32 | 8.59±0.46 |
| *Bacteroides* | 8.15±0.54 | 8.56±0.57 | 8.15±0.35 | 8.29±0.42 | 8.26±0.40 | 7.97±0.68 |
| Yeast | 6.29±0.43 | 6.51±0.42 | 6.56±0.45 ^b, c, *^ | 3.73±0.42 ^C^ | 3.62±0.49 ^C^ | 3.71±0.44 ^C^ |
| Day 42 | | | | | | |
| Anaerobic bacteria | 9.32±0.57 | 9.81±0.54 | 9.85±0.40 | 9.26±0.30 | 9.64±0.30 | 9.21±0.37 |
| *Lactobacillus* | 8.48±0.34 | 8.64±0.52 ^b, c, *^ | 9.19±0.46 ^b, c, *^ | 6.92±0.40 ^B, C^ | 7.17±0.69 ^B, C, *^ | 6.15±0.41 ^B, C, c^ |
| *Bifidobacterium* | 9.17±0.51 ^C, b, *^ | 9.70±0.43 ^b, *^ | 9.81±0.69 ^A, b, *^ | 8.33±0.53 ^A, B, C^ | 8.43±0.33 | 8.00±0.51 ^A, B, C^ |
| *Clostridium* | 5.26±0.37 ^b, c, *^ | 5.08±0.48 ^b, c, *^ | 4.42±0.52 ^b, c, *^ | 7.12±0.44 ^A, B, C, *^ | 7.10±0.42 ^A, B, C, *^ | 8.60±0.51 ^A, B, C, b, c^ |
| *Enterobacteriaceae* | 7.70±0.45 | 7.77±0.45 | 7.44±0.39 | 7.67±0.42 | 7.23±0.52 | 7.96±0.41 |
| *Escherichia coli* | 4.59±0.70 ^b, c, *^ | 4.21±0.40 ^b, c, *^ | 3.89±0.44 ^b, c, *^ | 6.56±0.45 ^A, B, C, *^ | 6.82±0.61 ^A, B, C^ | 7.87±0.35 ^A, B, C, b^ |
| *Enterococcus* | 8.40±0.45 | 8.19±0.45 | 8.64±0.63 | 8.30±0.42 | 8.33±0.55 | 8.78±0.39 |
| *Bacteroides* | 8.17±0.77 | 8.94±0.41 | 8.23±0.56 | 8.38±0.41 | 8.23±0.31 | 8.28±0.32 |
| Yeast | 7.04±0.43 ^b, c, *^ | 6.86±0.35 ^b, c, *^ | 7.30±0.36 ^b, c, *^ | 3.68±0.42 ^A, B, C^ | 3.70±0.41 ^A, B, C^ | 3.82±0.43 ^A, B, C^ |

**Table S4.** Counts of microorganisms which dominate in the content of the caecum of chickens fed with the synbiotic or probiotic-supplemented feed. The mean results from three repetitions for seven individuals from each group ± standard deviation (SD). Results significantly different from: ^*^ the control group; group of chickens fed with: ^A^ Synbiotic A; ^B^ Synbiotic B, ^C^ Synbiotic C; ^b^ BioPlus YC; ^c^ Cylactin; one-way ANOVA with post-hoc Tukey’s test (P < 0.05).

| Microorganism | Feed additives | | | | | |
| --- | --- | --- | --- | --- | --- | --- |
|  | **Synbiotic A** | **Synbiotic B** | **Synbiotic C** | **BioPlus YC** | **Cylactin** | **Without (Control)** |
|  | **log CFU g^-1^ (mean±SD)** | | | | | |
| Day 2 | | | | | | |
| Anaerobic bacteria | 7.37±0.27 | | | | | |
| *Lactobacillus* | 4.37±0.18 | | | | | |
| *Bifidobacterium* | 5.88±0.34 | | | | | |
| *Clostridium* | 5.04±0.28 | | | | | |
| *Enterobacteriaceae* | 4.60±0.18 | | | | | |
| *Escherichia coli* | 4.12±0.14 | | | | | |
| *Enterococcus* | 4.56±0.06 | | | | | |
| *Bacteroides* | 5.37±0.03 | | | | | |
| Yeast | 3.92±0.29 | | | | | |
| Day 7 | | | | | | |
| Anaerobic bacteria | 10.21±0.20 | 9.98±0.00 | 9.93±0.52 | 10.18±0.41 | 10.21±0.41 | 9.89±0.46 |
| *Lactobacillus* | 6.94±0.29 | 7.12±0.06 ^c^ | 7.70±0.31 ^c^ | 6.57±0.46 | 6.36±0.50 ^B, C^ | 6.32±0.36 |
| *Bifidobacterium* | 8.85±0.30 | 8.92±0.15 ^b, c, *^ | 9.00±0.22 | 8.17±0.48 ^B^ | 8.18±0.42 ^B^ | 8.04±0.26 ^B^ |
| *Clostridium* | 9.12±0.35 | 9.03±0.24 | 8.83±0.05 ^*^ | 9.29±0.52 | 9.31±0.52 | 9.39±0.04 ^C^ |
| *Enterobacteriaceae* | 8.18±0.12 | 8.28±0.47 | 8.26±0.38 | 8.13±0.59 | 8.09±0.62 | 8.22±0.03 |
| *Escherichia coli* | 6.73±0.17 ^*^ | 6.82±0.02^*^ | 6.29±0.13 ^*^ | 6.82±0.37 | 6.87±0.34 | 7.47±0.06 ^A, B, C^ |
| *Enterococcus* | 8.15±0.29 | 8.12±0.15 | 8.22±0.37 | 8.94±0.52 | 8.89±0.43 | 8.52±0.03 |
| *Bacteroides* | 9.23±0.23 | 9.51±0.01 | 9.07±0.32 | 9.19±0.66 | 9.03±0.40 | 9.44±0.10 |
| Yeast | 5.87±0.41 | 5.93±0.30 ^b, c, *^ | 5.83±0.34 ^b, c, *^ | 3.66±0.38 ^B, C^ | 3.84±0.41 ^B, C^ | 3.55±0.40 ^B, C^ |
| Day 14 | | | | | | |
| Anaerobic bacteria | 9.88±0.56 | 10.04±0.16 | 10.08±0.48 | 10.09±0.42 | 9.99±0.22 | 9.80±0.61 |
| *Lactobacillus* | 7.08±0.49 ^C^ | 7.32±0.41 | 8.01±0.56 ^A, b, c, *^ | 6.87±0.46 ^C^ | 6.84±0.61 ^C^ | 6.64±0.51 ^C^ |
| *Bifidobacterium* | 9.35±0.47 ^b, c, *^ | 9.49±0.64 ^b, c, *^ | 9.62±0.53 ^b, c, *^ | 8.36±0.43 ^A, B, C^ | 8.30±0.43 ^A, B, C^ | 8.09±0.49 ^A, B, C^ |
| *Clostridium* | 7.71±0.51 ^b, c, *^ | 7.38±0.46 ^b, c, *^ | 7.21±0.67 ^b, c, *^ | 9.03±0.61 ^A, B, C, *^ | 8.95±0.72 ^A, B, C, *^ | 9.48±0.77 ^A, B, C, b, c^ |
| *Enterobacteriaceae* | 8.35±0.51 | 8.21±0.47 | 8.12±0.54 | 8.62±0.43 | 8.54±0.45 | 8.50±0.63 |
| *Escherichia coli* | 5.81±0.38 ^b, *^ | 5.50±0.40 ^b, c, *^ | 4.88±0.40 ^b, c, *^ | 7.33±0.45 ^A, B, C^ | 7.14±0.31 ^B, C^ | 7.78±0.39 ^A, B, C^ |
| *Enterococcus* | 8.39±0.40 | 8.29±0.38 | 8.85±0.47 | 8.21±0.45 | 8.90±0.57 | 8.31±0.46 |
| *Bacteroides* | 9.01±0.36 | 9.57±0.59 | 9.44±0.52 | 9.28±0.63 | 9.16±0.34 | 9.29±0.39 |
| Yeast | 6.60±0.82 ^b, c, *^ | 6.48±0.30 | 6.27±0.36 ^b, c, *^ | 3.86±0.42 ^A, C^ | 3.89±0.37 ^A, C^ | 3.83±0.54 ^A, C^ |
| Day 28 | | | | | | |
| Anaerobic bacteria | 10.00±0.45 | 9.98±0.26 | 9.95±0.55 | 9.83±0.42 | 10.00±0.48 | 10.04±0.75 |
| *Lactobacillus* | 8.05±0.42 ^*^ | 8.17±0.51 ^b, c, *^ | 8.41±0.48 ^b, c, *^ | 7.01±0.48 ^B, C^ | 6.95±0.56 ^B, C^ | 6.70±0.47 ^A, B, C^ |
| *Bifidobacterium* | 9.57±0.38 ^b, c, *^ | 9.68±0.54 ^b, c, *^ | 9.81±0.58 ^b, c, *^ | 8.44±0.40 ^A, B, C^ | 8.35±0.45 ^A, B, C^ | 8.15±0.37 ^A, B, C^ |
| *Clostridium* | 6.75±0.29 ^*^ | 6.42±0.51 ^c, *^ | 6.23±0.33 ^c, *^ | 8.82±0.43 | 8.62±0.53 ^B, C, *^ | 9.56±0.48 ^A, B, C, c^ |
| *Enterobacteriaceae* | 8.19±0.38 | 8.47±0.60 | 8.21±0.41 | 8.75±0.56 | 8.53±0.33 | 8.73±0.56 |
| *Escherichia coli* | 4.58±0.35 ^b, c, *^ | 4.38±0.45 ^b, c, *^ | 4.15±0.42 ^b, c, *^ | 6.84±0.48 ^A, B, C, *^ | 7.04±0.42 ^A, B, C,*^ | 8.35±0.69 ^A, B, C, b, c^ |
| *Enterococcus* | 8.22±0.55 | 8.83±0.43 | 8.27±0.50 | 8.66±0.39 | 8.14±0.18 | 8.68±0.43 |
| *Bacteroides* | 8.85±0.43 | 8.56±0.38 | 8.37±0.39 | 8.48±0.38 | 8.72±0.56 | 8.82±0.95 |
| Yeast | 6.31±0.34 ^b, c, *^ | 6.35±0.44 ^b, c, *^ | 6.38±0.44 ^b, c, *^ | 4.13±0.41 ^A, B, C^ | 4.15±0.45 ^A, B, C^ | 4.03±0.36 ^A, B, C^ |
| Day 42 | | | | | | |
| Anaerobic bacteria | 10.08±0.61 | 10.04±0.41 | 10.19±0.48 | 9.98±0.45 | 9.86±0.34 | 10.03±0.45 |
| *Lactobacillus* | 8.21±0.56 ^C, b, *^ | 8.39±0.44 ^b, *^ | 9.01±0.48 ^A, b, c, *^ | 7.14±0.56 ^A, B, C^ | 7.30±0.35 ^C^ | 7.07±0.43 ^A, B, C^ |
| *Bifidobacterium* | 9.69±0.45 ^b, c, *^ | 9.84±0.62 ^b, c, *^ | 9.94±0.49 ^b, c, *^ | 8.46±0.48 ^A, B, C^ | 8.39±0.44 ^A, B, C^ | 8.18±0.45 ^A, B, C^ |
| *Clostridium* | 6.28±0.35 ^b, *^ | 6.00±0.43 ^b, *^ | 5.60±0.43 ^b, c, *^ | 8.30±0.49 ^A, B, C, *^ | 8.11±0.43 ^C, *^ | 9.43±0.49 ^A, B, C, b, c^ |
| *Enterobacteriaceae* | 8.51±0.44 | 8.75±0.62 | 8.45±0.43 | 8.68±0.43 | 8.21±0.46 | 8.48±0,43 |
| *Escherichia coli* | 4.46±0.39 ^b, c, *^ | 4.15±0.41^b, c, *^ | 4.04±0.41 ^b, c, *^ | 6.48±0.38 ^A, B, C, *^ | 6.17±0.44 ^A, B, C, *^ | 8.32±0.46 ^A, B, C, b, c^ |
| *Enterococcus* | 8.30±0.55 | 8.42±0.44 | 8.73±0.66 | 8.53±0.36 | 8.87±0.40 | 8.52±0.46 |
| *Bacteroides* | 9.30±0.55 | 9.43±0.48 | 9.76±0.45 | 9.29±0.41 | 9.25±0.43 | 9.20±0.52 |
| Yeast | 7.16±0.45 ^b, c, *^ | 7.27±0.47 ^b, c, *^ | 7.42±0.41 ^b, c, *^ | 4.26±0.39 ^A, B, C^ | 4.54±0.43 ^A, B, C^ | 4.20±0.56 ^A, B, C^ |

**Table S5.** Counts of microorganisms which dominate in the excreta of chickens fed with the synbiotic or probiotic-supplemented feed. The mean results from three repetitions for seven individuals from each group ± standard deviation (SD). Results significantly different from: ^*^ the control group; group of chickens fed with: ^A^ Synbiotic A; ^B^ Synbiotic B, ^C^ Synbiotic C; ^b^ BioPlus YC; ^c^ Cylactin; one-way ANOVA with post-hoc Tukey’s test (P < 0.05).

| Microorganism | Feed additives | | | | | |
| --- | --- | --- | --- | --- | --- | --- |
|  | **Synbiotic A** | **Synbiotic B** | **Synbiotic C** | **BioPlus YC** | **Cylactin** | **Without (Control)** |
|  | **log CFU g^-1^ (mean±SD)** | | | | | |
| Day 2 | | | | | | |
| Anaerobic bacteria | 6.67±0.06 | | | | | |
| *Lactobacillus* | 4.18±0.07 | | | | | |
| *Bifidobacterium* | 5.67±0.06 | | | | | |
| *Clostridium* | 4.92±0.12 | | | | | |
| *Enterobacteriaceae* | 4.97±0.36 | | | | | |
| *Escherichia coli* | 4.69±0.02 | | | | | |
| *Enterococcus* | 5.23±0.01 | | | | | |
| *Bacteroides* | 5.58±0.14 | | | | | |
| Yeast | 5.09±0.03 | | | | | |
| Day 7 | | | | | | |
| Anaerobic bacteria | 10.32±0.12 | 9.94±0.01 | 9.94±0.57 | 9.91±0.53 | 10.10±0.44 | 9.90±0.24 |
| *Lactobacillus* | 7.02±0.21 ^C^ | 7.42±0.29 | 7.89±0.08 ^A, b, c, *^ | 6.27±0.43 ^C^ | 6.20±0.54 ^C^ | 6.08±0.29 ^C^ |
| *Bifidobacterium* | 8.62±0.03 | 8.81±0.14 ^b^ | 9.07±0.18 | 8.40±0.58 ^B^ | 8.34±0.46 | 8.13±0.32 |
| *Clostridium* | 8.23±0.10 | 8.13±0.23 | 7.79±0.04 ^b^ | 8.26±0.61 ^C^ | 8.33±0.47 | 8.53±0.42 |
| *Enterobacteriaceae* | 8.53±0.05 | 8.62±0.41 | 8.64±0.10 | 8.14±0.36 | 8.25±0.38 | 8.90±0.28 |
| *Escherichia coli* | 6.15±0.12 | 6.18±0.24 | 5.92±0.23 | 6.82±0.38 | 6.93±0.39 | 7.12±0.29 |
| *Enterococcus* | 9.05±0.37 | 9.19±0.04 | 9.18±0.23 | 9.14±0.59 | 9.29±0.60 | 9.15±0.10 |
| *Bacteroides* | 9.52±0.31 | 9.46±0.38 | 9.62±0.33 | 9.37±0.48 | 9.41±0.49 | 9.53±0.25 |
| Yeast | 5.41±0.01 ^b, c, *^ | 5.84±0.42 | 5.11±0.18 ^b, c, *^ | 4.41±0.50 ^A, C^ | 4.54±0.47 ^A, C^ | 4.10±0.14 ^A, C^ |
| Day 14 | | | | | | |
| Anaerobic bacteria | 10.07±0.57 | 9.99±0.84 | 10.00±0.71 | 9.93±0.49 | 9.93±0.67 | 10.02±0.86 |
| *Lactobacillus* | 8.88±0.44 ^b, c, *^ | 9.01±0.46 ^b, c, *^ | 9.14±0.57 ^b, c, *^ | 6.56±0.38 ^A, B, C^ | 6.52±0.39 ^A, B, C^ | 6.39±0.35 ^A, B, C^ |
| *Bifidobacterium* | 8.74±0.42 | 9.11±0.44 | 9.36±0.48 ^c, *^ | 8.58±0.40 | 8.61±0.51 ^C^ | 8.48±0.41^C^ |
| *Clostridium* | 7.76±0.49 ^b, c, *^ | 7.27±0.37 ^b, c, *^ | 7.05±0.49 ^b, c, *^ | 8.37±0.84 ^A, B, C, *^ | 8.50±0.70 ^A, B, C, *^ | 9.16±0.81 ^A, B, C, b, c^ |
| *Enterobacteriaceae* | 8.18±0.40 | 8.57±0.59 | 8.48±0.60 | 8.61±0.33 | 8.38±0.22 | 8.75±0.57 |
| *Escherichia coli* | 5.90±0.45 ^C, *^ | 5.55±0.37^*^ | 4.64±0.21 ^A, b, c, *^ | 6.71±0.32^C^ | 6.89±0.40 ^C^ | 7.35±0.47 ^A, B, C^ |
| *Enterococcus* | 9.56±0.59 | 9.87±0.52 | 9.61±0.71 | 9.57±0.41 | 9.46±0.57 | 9.77±0.36 |
| *Bacteroides* | 9.36±0.55 | 9.58±0.61 | 9.79±0.64 | 9.73±0.66 | 9.60±0.26 | 9.74±0.43 |
| Yeast | 6.88±0.39 ^b, c, *^ | 6.85±0.38 ^b, c, *^ | 6.99±0.45 ^b, c, *^ | 4.38±0.51 ^A, B, C^ | 4.62±0.43 ^A, B, C^ | 4.16±0.63 ^A, B, C^ |
| Day 28 | | | | | | |
| Anaerobic bacteria | 10.11±0.42 | 9.94±0.42 | 10.11±0.58 | 9.99±0.46 | 9.82±0.80 | 10.01±0.47 |
| *Lactobacillus* | 9.34±0.41 ^b, c, *^ | 9.52±0.44 ^b, c, *^ | 9.72±0.57 ^b, c, *^ | 7.37±0.36 ^A, B, C^ | 7.47±0.60 ^A, B, C^ | 6.97±0.44 ^A, B, C^ |
| *Bifidobacterium* | 9.38±0.40 ^C^ | 9.54±0.52 ^c, *^ | 9.89±0.88 ^A, b, c, *^ | 8.94±0.43 ^C^ | 8.79±0.67 ^B, C^ | 8.71±0.47 ^B, C^ |
| *Clostridium* | 6.43±0.51 ^b^ | 6.00±0.81 ^b^ | 5.70±0.40 ^b^ | 8.22±0.59 ^A, B, C^ | 8.31±0.43 | 9.38±0.43 |
| *Enterobacteriaceae* | 8.41±0.64 | 8.16±0.46 | 8.21±0.49 | 8.24±0.57 | 8.36±0.36 | 8.90±0.87 |
| *Escherichia coli* | 5.15±0.43 ^b, c, *^ | 5.00±0.48 ^b, c, *^ | 4.85±0.49 ^b, c, *^ | 6.65±0.36 ^A, B, C, *^ | 6.76±0.24 ^A, B, C, *^ | 8.48±0.48 ^A, B, C, b, c^ |
| *Enterococcus* | 9.55±0.89 | 9.22±0.43 | 9.42±0.59 | 9.39±0.39 | 9.19±0.58 | 9.84±0.34 |
| *Bacteroides* | 9.17±0.41 | 9.15±0.38 | 9.34±0.76 | 8.97±0.39 | 8.88±0.73 | 9.00±0.46 |
| Yeast | 6.22±0.54 ^b, c, *^ | 6.49±0.59 ^b, c, *^ | 6.33±0.48 ^b, C, *^ | 4.29±0.47 ^A, B, C^ | 4.35±0.38 ^A, B, C^ | 4.12±0.55 ^A, B, C^ |
| Day 42 | | | | | | |
| Anaerobic bacteria | 9.95±0.46 | 9.91±1.08 | 10.18±0.48 | 10.01±0.39 | 9.91±0.43 | 9.87±0.77 |
| *Lactobacillus* | 9.51±0.41 ^b, c, *^ | 9.70±0.65 ^b, c, *^ | 9.89±0.73 ^b, c, *^ | 7.70±0.41 ^A, B, C^ | 7.62±0.50 ^A, B, C^ | 7.41±0.43 ^A, B, C^ |
| *Bifidobacterium* | 9.57±0.42 ^*^ | 9.66±0.66 ^c, *^ | 9.96±0.46 | 9.06±0.35 | 9.14±0.65 ^B^ | 8.81±0.50 ^A, B^ |
| *Clostridium* | 6.10±0.44 ^b, c, *^ | 5.72±0.49 ^b, c, *^ | 5.48±0.43 ^b, c, *^ | 7.70±0.44 ^A, B, C, *^ | 7.52±0.43 ^A, B, C, *^ | 9.24±0.48 ^A, B, C, b, c^ |
| *Enterobacteriaceae* | 8.23±0.67 | 8.75±0.50 | 8.22±0.43 | 8.19±0.44 | 8.21±0.44 | 8.62±0.58 |
| *Escherichia coli* | 4.39±0.45 ^b, *^ | 4.24±0.47 ^b, c, *^ | 4.05±0.67 ^b, c, *^ | 6.43±0.42 ^A, B, C, *^ | 6.60±0.37 ^B, C^ | 8.44±0.35 ^A, B, C, b^ |
| *Enterococcus* | 9.57±0.32 | 9.79±1.06 | 9.26±0.44 | 9.24±0.39 | 9.50±0.53 | 9.63±0.76 |
| *Bacteroides* | 9.16±0.51 | 9.10±0.76 | 9.24±0.41 | 9.27±0.41 | 9.17±0.38 | 9.36±0.63 |
| Yeast | 6.68±0.43 ^b, c, *^ | 6.82±0.44 ^b, c, *^ | 7.00±0.47 ^b, c, *^ | 4.57±0.46 ^A, B, C^ | 4.32±0.38 ^A, B, C^ | 4.30±0.51 ^A, B, C^ |

**Table S6.** Changes in lactic acid, SCFA and BCFA concentrations in the excreta of chickens fed with the synbiotic or probiotic-supplemented feed. The mean results from three repetitions for seven individuals from each group ± standard deviation (SD). Results significantly different from: ^*^ the control group; group of chickens fed with: ^A^ Synbiotic A; ^B^ Synbiotic B, ^C^ Synbiotic C; ^b^ BioPlus YC; ^c^ Cylactin; one-way ANOVA with post-hoc Tukey’s test (P < 0.05).

|  | Day | Feed additives | | | | | |
| --- | --- | --- | --- | --- | --- | --- | --- |
|  |  | **Synbiotic A** | **Synbiotic B** | **Synbiotic C** | **BioPlus YC** | **Cylactin** | **Without (Control)** |
|  |  | **The mean concentration of acid (µM g^-1^ ±SD)** | | | | | |
|  | **Lactic acid (LA)** | | | | | | |
|  | 2 | 17.86±1.26 | | | | | |
|  | 42 | 22.61±2.35 ^C, *^ | 25.29±4.44 ^b, *^ | 27.32±3.66 ^A, b, c, *^ | 20.71±2.17 ^B, C, *^ | 21.56±3.90 ^C, *^ | 17.90±1.26 ^A, B, C, b, c^ |
| SCFA | **Acetic acid** | | | | | | |
|  | 2 | 5.74±0.90 | | | | | |
|  | 42 | 13.50±2.67 ^*^ | 13.33±1.08 ^*^ | 13.81±1.98 ^*^ | 11.61±1.92 ^*^ | 12.72±1.40 ^*^ | 6.57±0.73 ^A, B, C, b, c^ |
|  | **Propionic acid** | | | | | | |
|  | 2 | 0.46±0.19 | | | | | |
|  | 42 | 0.89±0.22 ^b, *^ | 0.94±0.22 ^b, *^ | 0.97±0.21 ^b, *^ | 0.58±0.17 ^A, B, C^ | 0.70±0.36 | 0.47±0.24 ^A, B, C^ |
|  | **Butyric acid** | | | | | | |
|  | 2 | 0.49±0.03 | | | | | |
|  | 42 | 1.89±0.31 ^b, c, *^ | 2.15±0.60 ^b, c, *^ | 2.48±0.96 ^b, c, *^ | 1.21±0.33 ^A, B, C, *^ | 1.20±0.40 ^A, B, C, *^ | 0.47±0.10 ^A, B, C, b, c, *^ |
|  | **Valeric acid** | | | | | | |
|  | 2 | 0.20±0.07 | | | | | |
|  | 42 | 0.28±0.10 ^C^ | 0.34±0.08 ^*^ | 0.40±0.10 ^A, *^ | 0.28±0.16 | 0.27±0.14 | 0.20±0.08 ^B, C^ |
|  | **Formic acid** | | | | | | |
|  | 2 | 1.53±0.15 | | | | | |
|  | 42 | 2.43±0.23 ^C, b, c, *^ | 2.72±0.28 ^b, c, *^ | 2.99±0.34 ^A, b, c, *^ | 1.87±0.18 ^B, C, *^ | 1.92±0.15 ^C, *^ | 1.33±0.10 ^A, B, C, b, c^ |
|  | **The sum of SCFA** | | | | | | |
|  | 2 | 8.42±0.93 | | | | | |
|  | 42 | 18.99±2.90 ^b, *^ | 19.48±1.25 ^b, c, *^ | 20.65±1.89 ^b, c, *^ | 15.55±2.02 ^A, B, C, *^ | 16.81±1.66 ^B, C, *^ | 9.04±0.60 ^A, B, C, b, c^ |
| BCFA | **Isobutyric acid** | | | | | | |
|  | 2 | 0.30±0.11 | | | | | |
|  | 42 | 0.15±0.09 ^c, *^ | 0.14±0.07 ^c, *^ | 0.12±0.07 ^b, c, *^ | 0.23±0.09 ^C^ | 0.27±0.07 ^A, B, C^ | 0.30±0.11 ^A, B, C^ |
|  | **Isovaleric acid** | | | | | | |
|  | 2 | 0.12±0.06 | | | | | |
|  | 42 | 0.06±0.02 ^c, *^ | 0.07±0.04 | 0.06±0.03 ^*^ | 0.09±0.02 | 0.10±0.04 ^A^ | 0.11±0.03 ^A, C^ |
|  | **The sum of BCFA** | | | | | | |
|  | 2 | 0.42±0.16 | | | | | |
|  | 42 | 0.21±0.10 ^c, *^ | 0.21±0.11 ^c, *^ | 0.18±0.08 ^b, c, *^ | 0.32±0.08 ^C^ | 0.37±0.07 ^A, B, C^ | 0.41±0.14 ^A, B, C^ |
| LA: SCFA:BCFA | 2 | 67:31:2 | | | | | |
|  | 42 | 54:45:1 | 56:43:1 | 56:43:1 | 57:42:1 | 56:43:1 | 65:33:2 |
